# Supplementary figures and images for: Rfx2 Stabilizes Foxj1 Binding at Chromatin Loops to Enable Multiciliated Cell Gene Expression
Source: PLoS Genet. 2017 Jan 19;13(1):e1006538. doi: 10.1371/journal.pgen.1006538 (PMC5245798; doi:10.1371/journal.pgen.1006538)

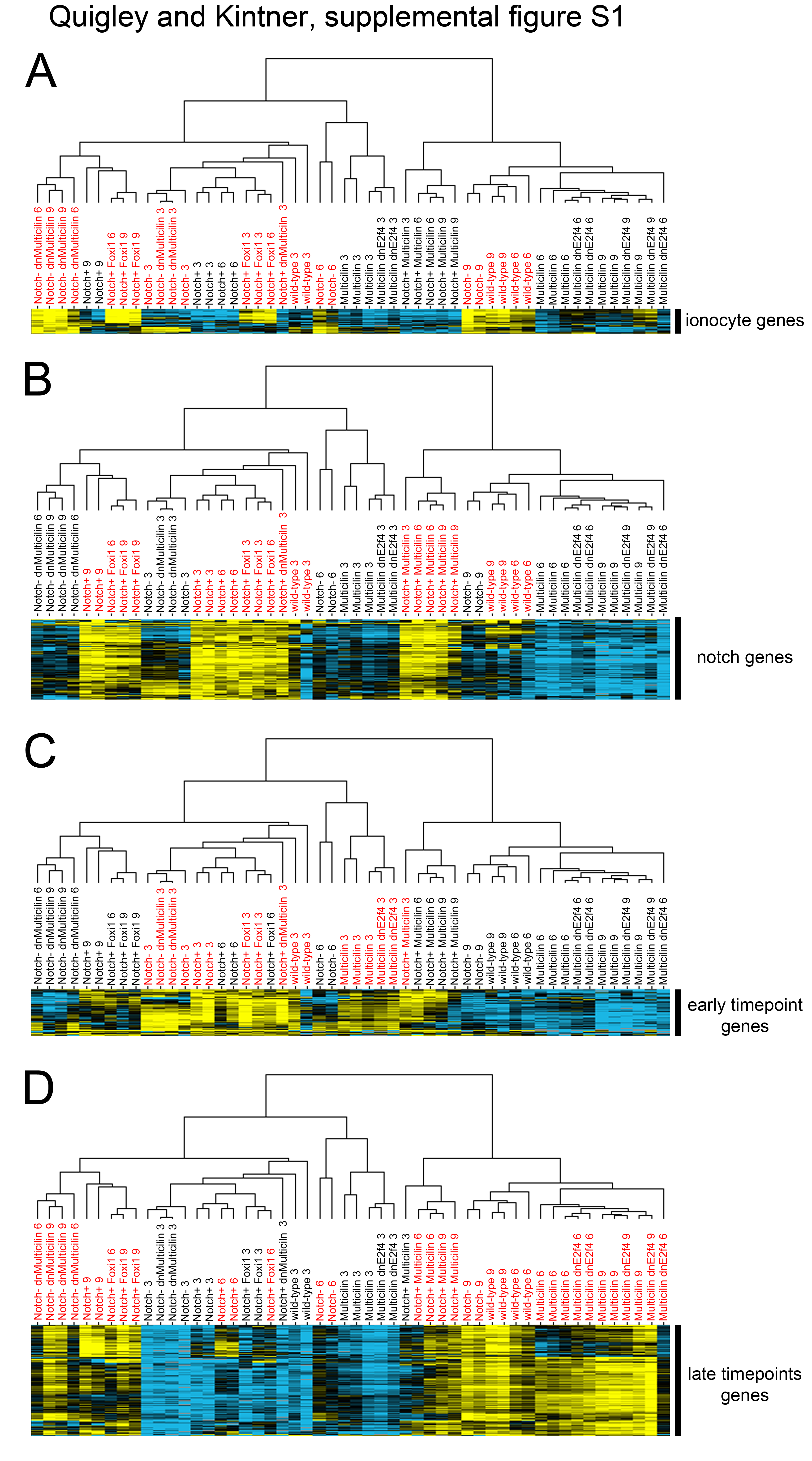

Supplement: S1 Fig — RNAseq analysis was carried out on epithelial progenitors that were isolated from embryos injected with RNA to increase (Notch-icd, labeled as Notch+ [83,84]) or decrease (DNA-binding mutant of Suppressor of Hairless or DBM, labeled as Notch- [18,84]) Notch signaling, to increase (Multicilin, [19]) or decrease (dominant-negative Multicilin or dnMulticilin, [19] Multicilin activity, to increase (Foxi1, [67]) Foxi1 activity, or to inhibit E2f4 (E2f4ΔCT, [40] activity. Samples were collected at 3, 6 or 9 hour timepoints, corresponding to the equivalent of stages 13, 16, and 18, respectively, and hierarchical clustering performed on both samples and expression. (A-D) Shown are groups of genes clustered by similarity of expression. Each group shows increased expression corresponding to subsets of experimental treatment. In each case, these treatments are in red (e.g., in (A), Notch-, Notch- Foxi1+, Notch- dnMulticilin, and wild-type treatments all have more ionocytes than Notch+ or Multicilin treatments). (A) Genes clustered by similarity of expression found to be increased in treatments promoting ionocytes (also see S2 Table). (B) Genes clustered by similarity of expression found to be increased in treatments with more Notch signaling (also see S3 Table). (C) Genes clustered by similarity of expression found to be increased in treatments harvested at the earliest timepoint (3 hours after mid-stage 11, or roughly stage 13; also see S4 Table). (D) Genes clustered by similarity of expression found to be increased in treatments harvested at the later timepoints ((6 and 9 hours after mid-stage 11, or roughly stages 16 and 18; also see S5 Table). This group contains genes associated with the differentiation of goblet and small secretory cells. (TIF) [file pgen.1006538.s001.tif]

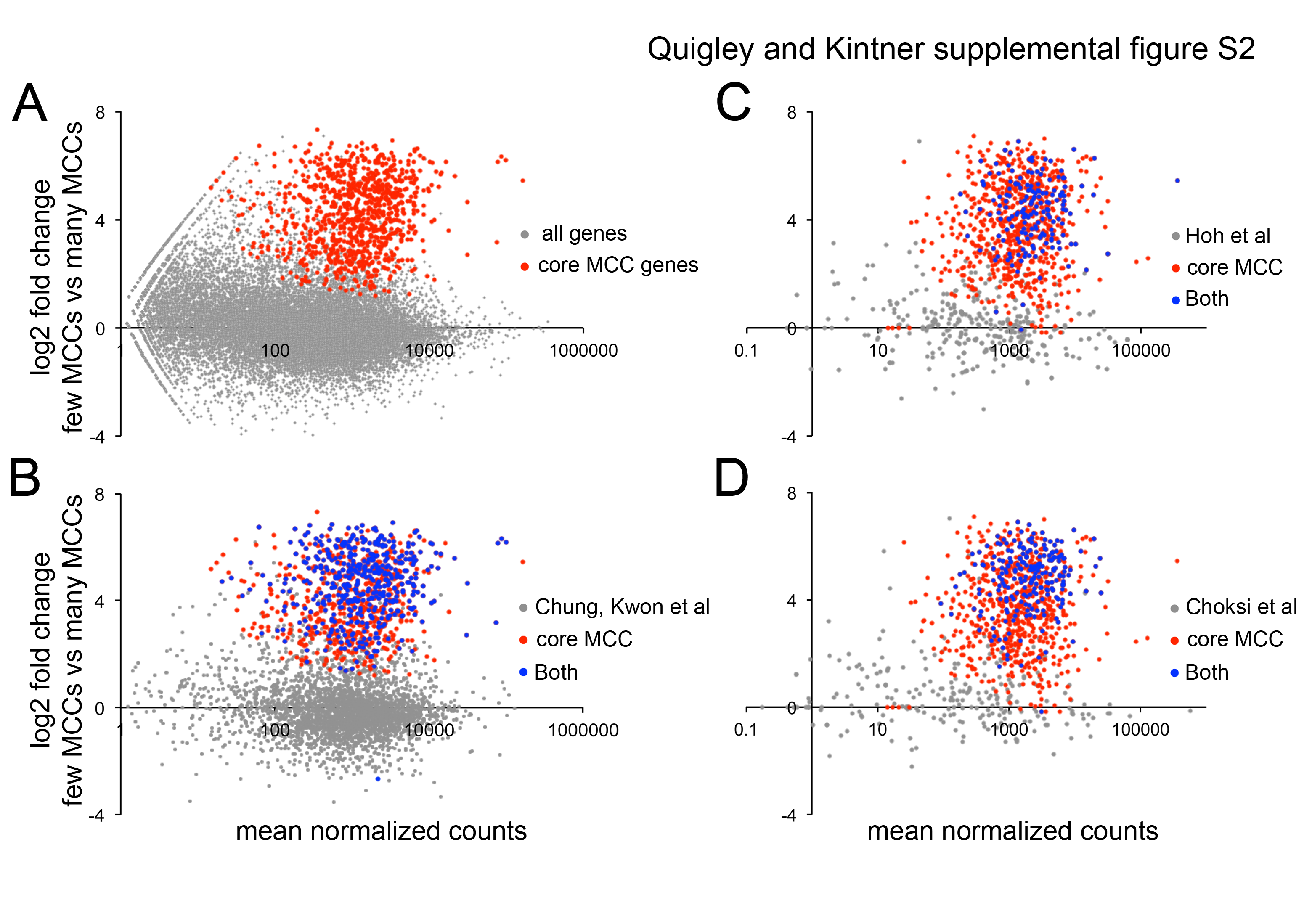

Supplement: S2 Fig — (A) MA plot (log ratios vs. mean average) of all genes expressed in dissected X. laevis ectoderm at the 9 hour timepoint. X axis is normalized counts per gene and the Y axis is log2-fold change of each gene in a comparison between progenitors injected with Notch-icd (“few MCCs”) versus progenitors injected with Notch-icd and Multicilin (“many MCCs”). Core MCC genes labeled in red as determined in Fig 1C, all other X. laevis genes labeled in gray. (B) Data from (A) but showing only genes from a comparison between in our core MCC list and a multiliciated cell transcriptome obtained by knocking down Rfx2 in X. laevis [22]. Shown are genes only found in our core MCC list (red), genes only found from [22], or genes found in both lists. (C,D) Many X. laevis genes have two paralogs owing to pseudotetraploidy. In order to compare our data to diploid organisms, we collapsed RNAseq counts from paralogs into a single gene. (C) Data from (A) but showing only genes in a comparison between our core MCC list and a motile cilia transcriptome obtained by sorting Foxj1+ cells from mouse tracheal epithelial cultures [21]. Shown is a similar comparison as (B) and (C). (D) Data from (A) but showing only genes from a comparison between our core MCC list and a motile cilia transcriptome obtained by overexpressing Foxj1 in zebrafish embryos [20]. Shown are genes only found in our core MCC list (red), genes only found from [20], or genes found in both lists. Please note that as we have collapsed X. laevis L and S forms into a single transcript in (C) and (D), the plots will look slightly different. (TIF) [file pgen.1006538.s002.tif]

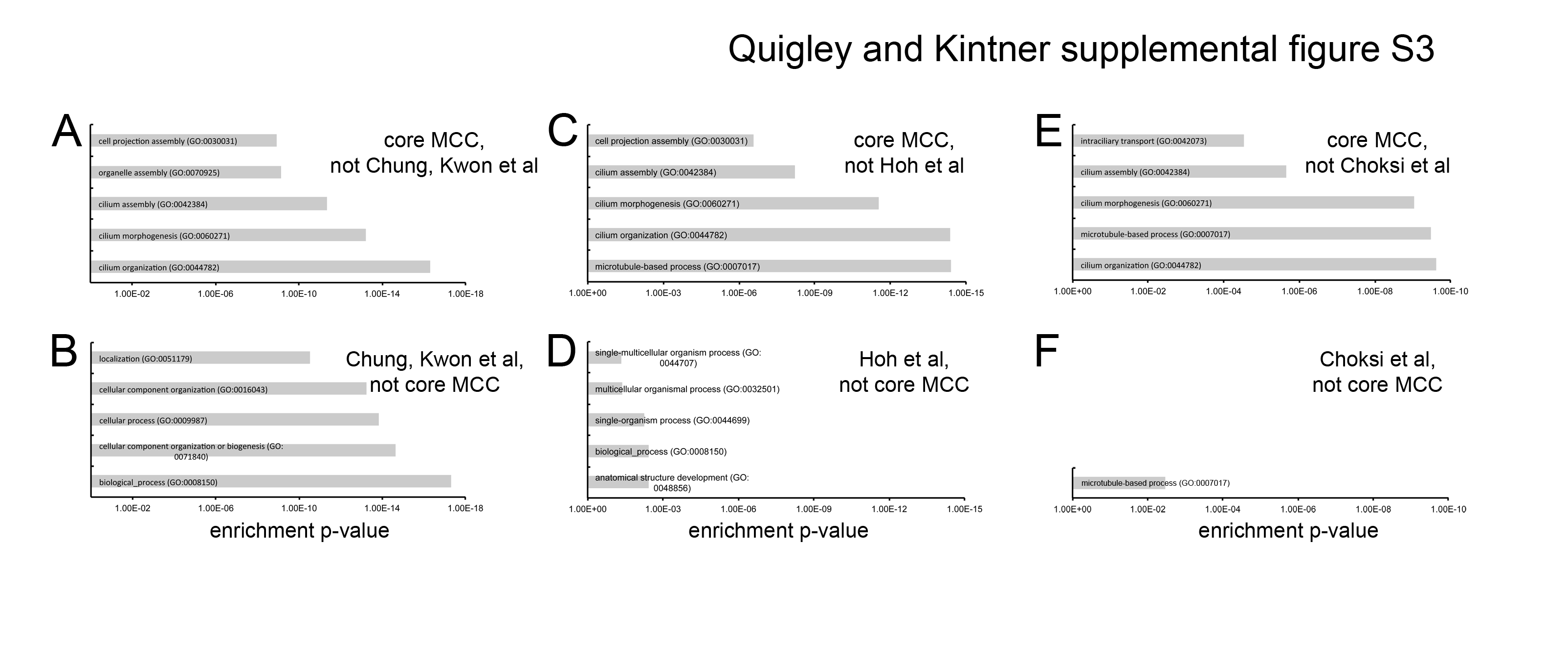

Supplement: S3 Fig — (A,B) GO term enrichment of genes shown in S2B Fig that are present on our core MCC list but not regulated by Rfx2 (A), or vice versa (B). (C,D) GO term enrichment of genes shown in S2C Fig that are present on our core MCC list but not expressed in Foxj1+ mouse lung cells (C) or vice versa (D). (E,F) GO term enrichment of genes that are shown in S2D Fig present on our core MCC list but not induced in Zebrafish by Foxj1 overexpression (E) or vice versa (F). (TIF) [file pgen.1006538.s003.tif]

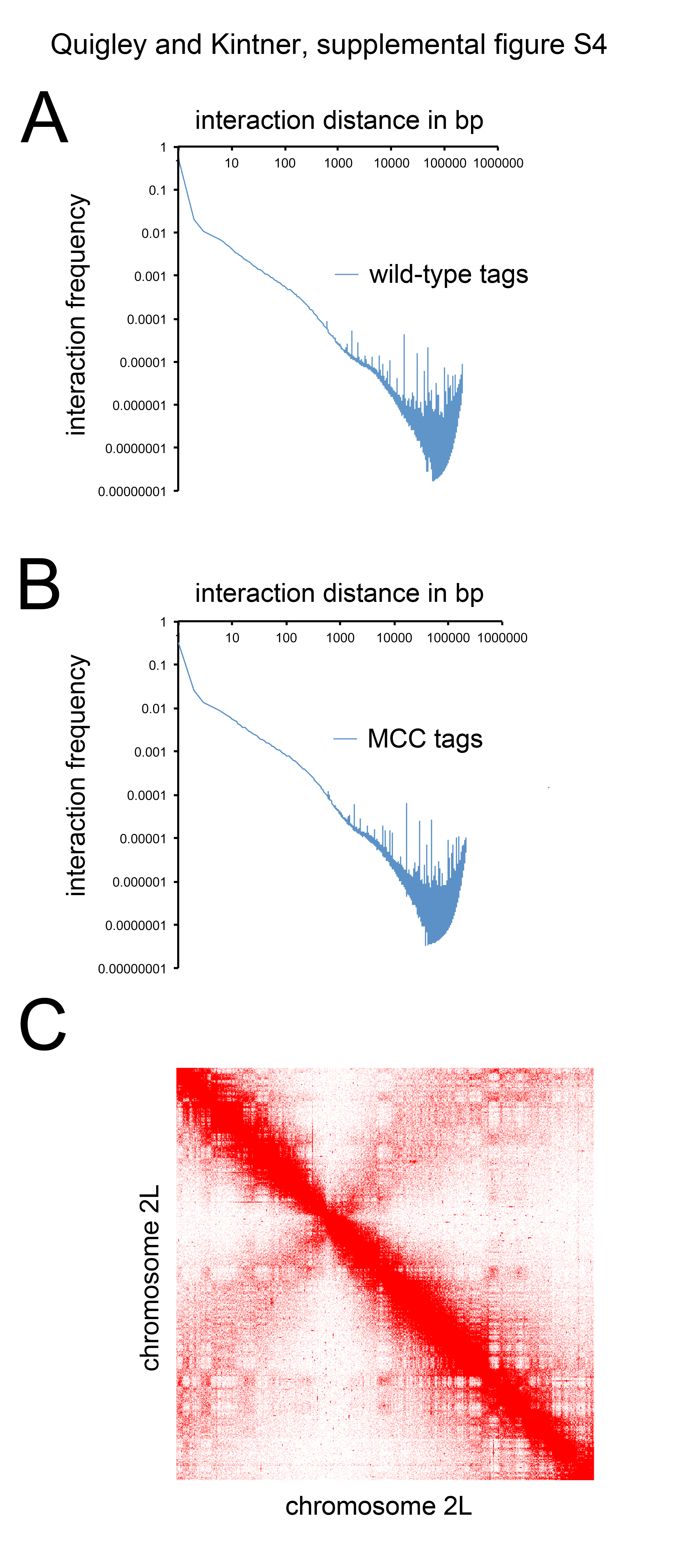

Supplement: S4 Fig — (A,B) Tethered conformation capture performed by binding DNA-protein complexes to a fixed substrate followed by digestion by restriction enzymes (in this case, MboI) and proximity ligation. Interaction frequencies are reported to drop as a function of linear genomic distance [8,25]; the majority of proximity ligation events are the result of immediately neighboring sequence, and not larger 3D structure. Moreover, interchromosomal interactions are thought to be quite rare and strong enrichment for these interactions in the data is suggestive of spurious ligations. We saw high frequencies of local interactions and few interchromosomal interactions in the X. laevis genome (A,B). Data are paired-end sequencing tags from a total of 139,003,458 uniquely mapped reads. (C) Raw interaction matrix of X. laevis chromosome 2L showing contacts across loci. (TIF) [file pgen.1006538.s004.tif]

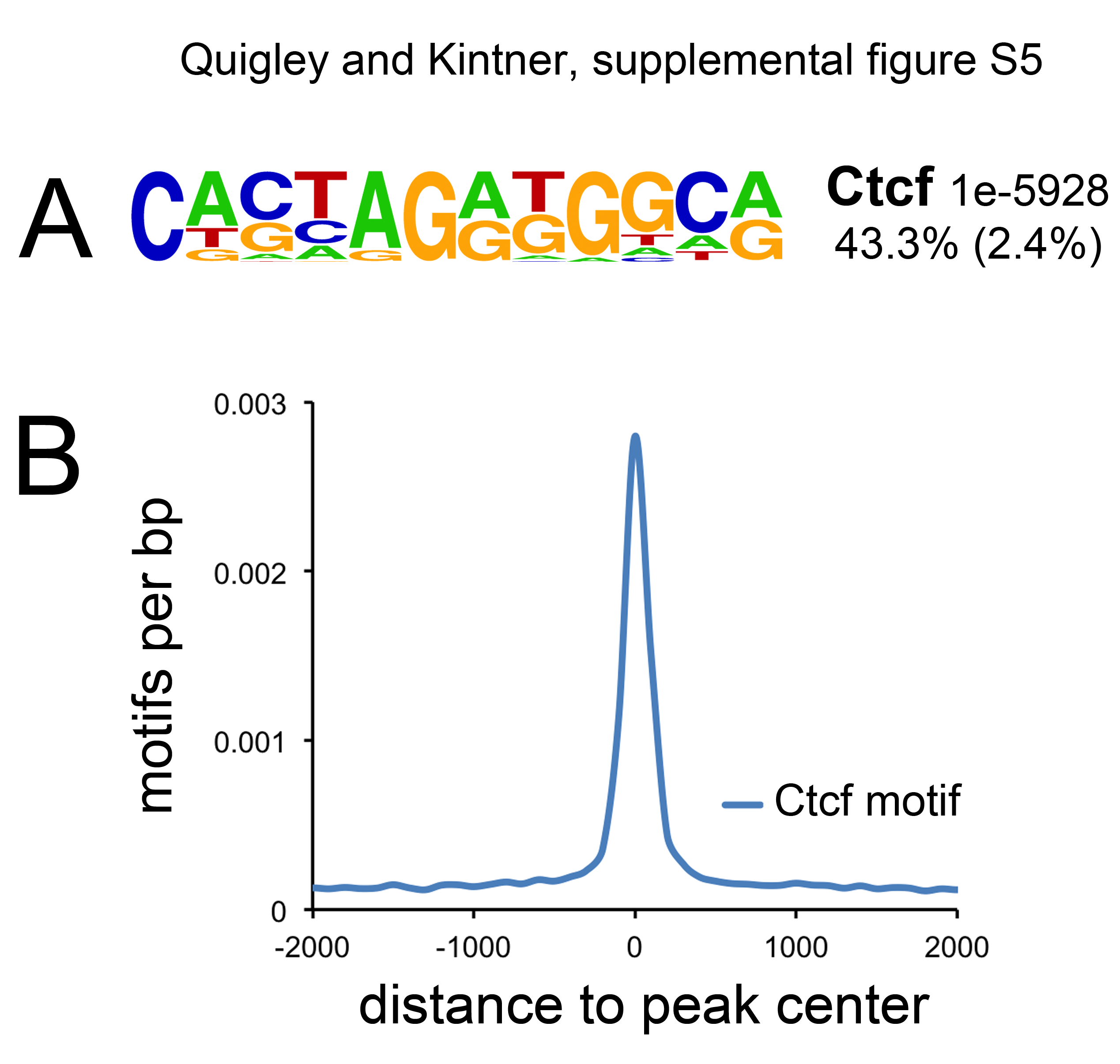

Supplement: S5 Fig — (A) Top de novo motif hit from all Rad21 peaks. Top line of label is transcription factor family binding the motif and p-value; second line of label is frequency of motif in peaks versus background frequency (background frequency is in parentheses). (B) Frequency and position of top motif in all Rad21 peaks. (TIF) [file pgen.1006538.s005.tif]

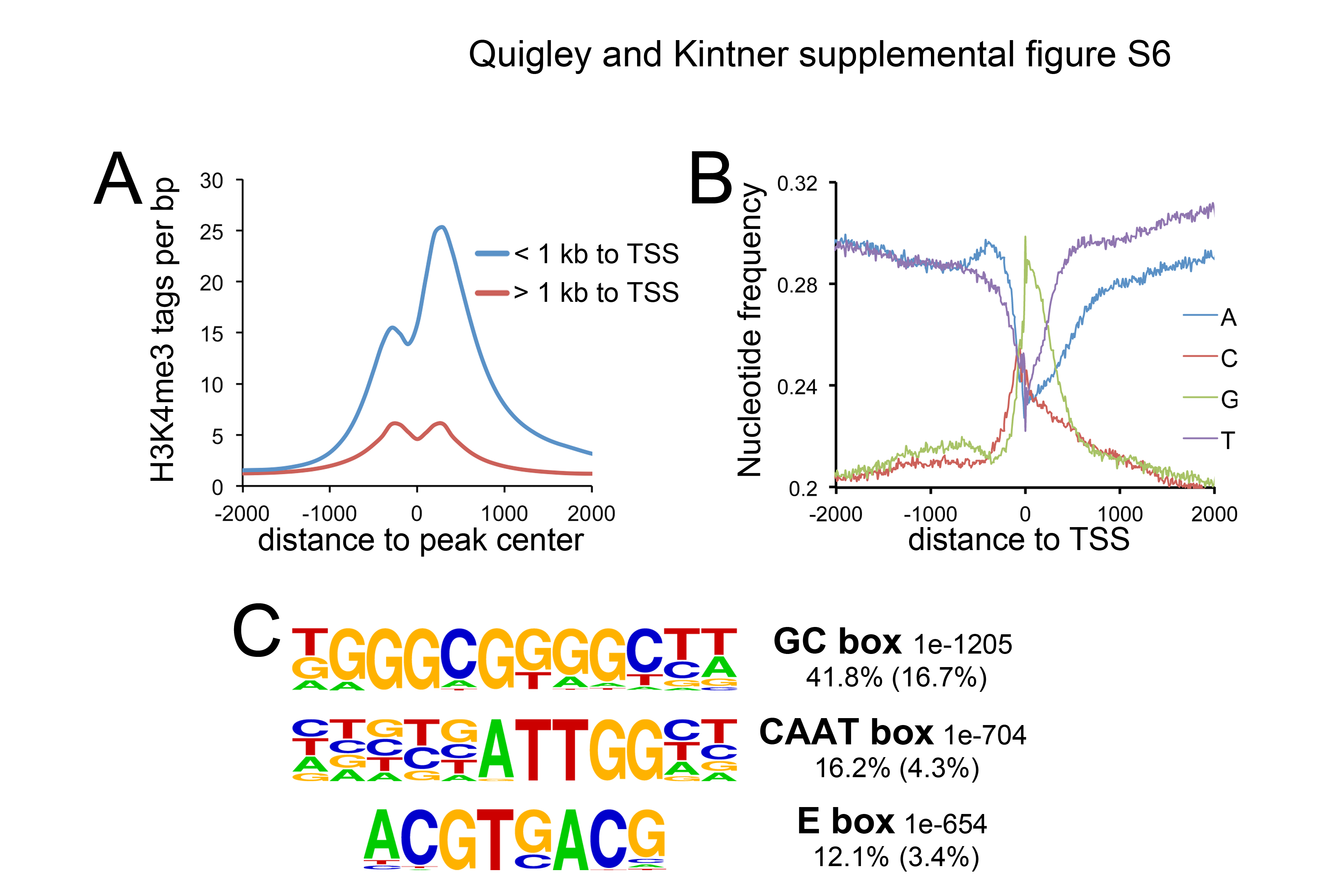

Supplement: S6 Fig — (A) Tag counts obtained from H3K4me3 ChIPseq on progenitors overlapping or not overlapping the annotated TSS of X. laevis genes (Mayball models, [22]). Note dip signifying nucleosome-free region at center of peak. (B) Nucleotide frequencies around promoter peak centers. Note GC-rich bias around the TSS (Louie et al., 2011., also see S10 Fig). (C) Top de novo motifs identified in sequences called as peaks in ChIPseq analysis of all H3K4me3-positive promoters in epithelial progenitors. Top line of label is transcription factor family binding the motif and p-value; second line of label is frequency of motif in peaks versus background frequency (background frequency obtained by searching GC-matched genomic sequence is in parentheses). (TIF) [file pgen.1006538.s006.tif]

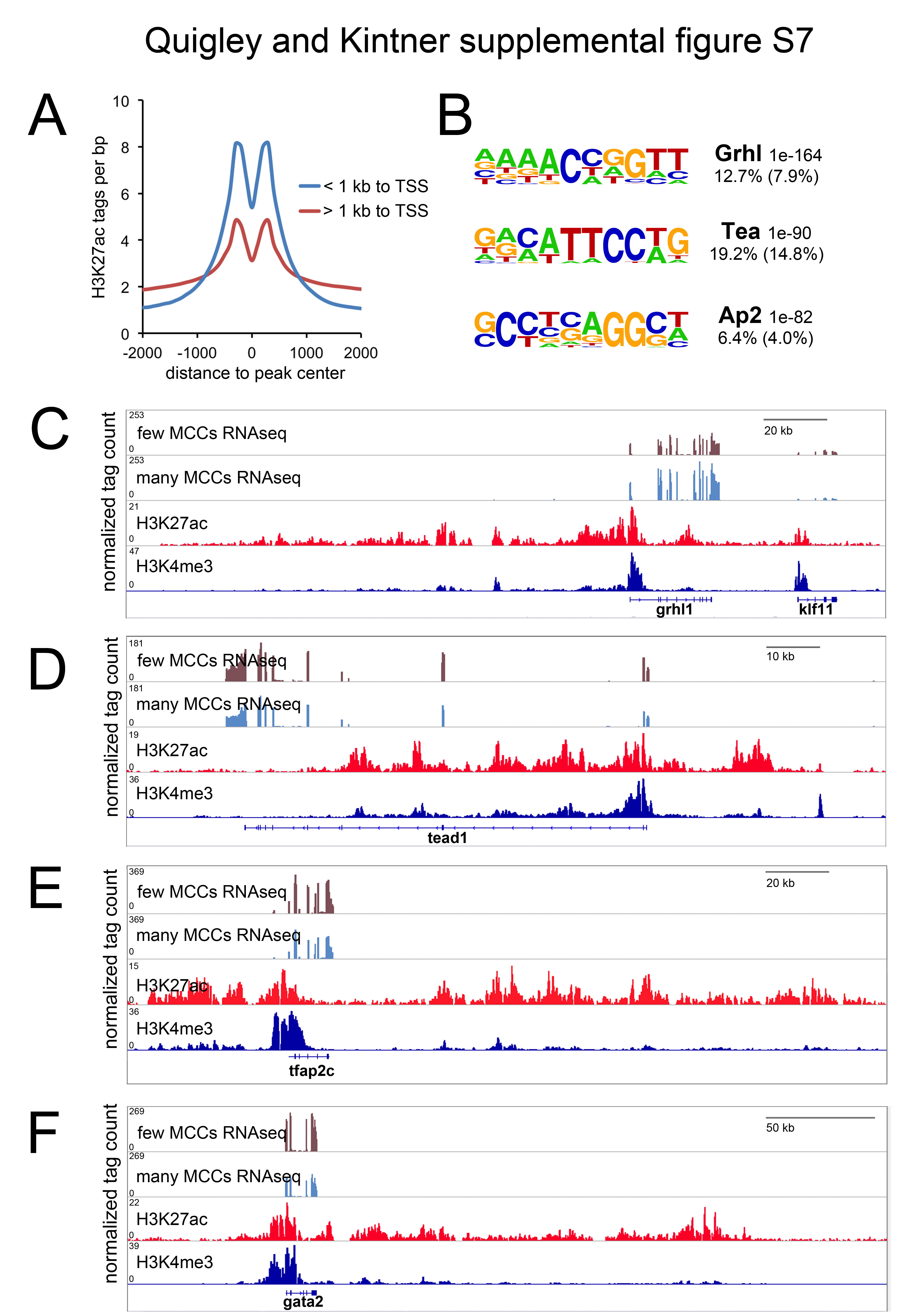

Supplement: S7 Fig — (A) H3K27ac tag counts around peak centers of confirmed X. laevis promoters and distal sites obtained by ChIPseq performed on isolated epithelial progenitors. Note nucleosome-free region at dip in center of peak. (B) Top de novo motif hits from distal H3K27ac peaks. Top line of label is transcription factor family binding the motif and p-value; second line of label is frequency of motif in peaks versus background frequency (background frequency is in parentheses). (C-F) Select superenhancers defined by the strategy described in [85]. Note superenhancers around key transcription factors (C-E) that recognize the most-enriched motifs from all enhancers (B). (F) Superenhancer around gata2, hinting at an underappreciated role for gata transcription factors in epithelial differentiation. (TIF) [file pgen.1006538.s007.tif]

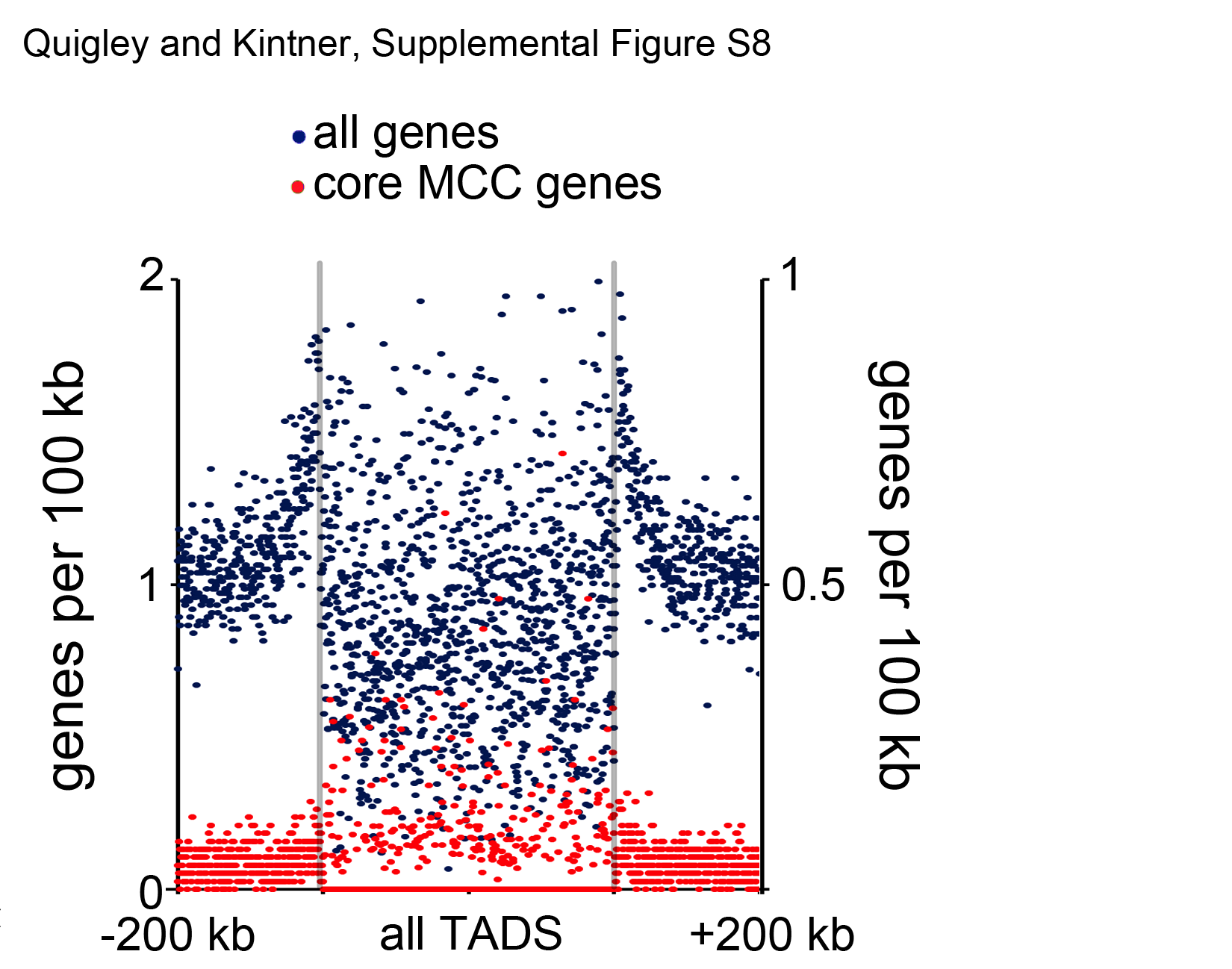

Supplement: S8 Fig — Metagene plot showing the distribution of core MCC genes relative to all genes in TADs. Note far fewer MCC genes within TADs: bins lacking MCC genes display as dots at bottom of Y axis. Domain region is in the center with boundaries marked, and all domains are normalized to the same size, whereas flanking areas are 200 kb upstream and downstream of those domain boundaries. Each quartile is broken into 175 bins, and each dot denotes one bin. (TIF) [file pgen.1006538.s008.tif]

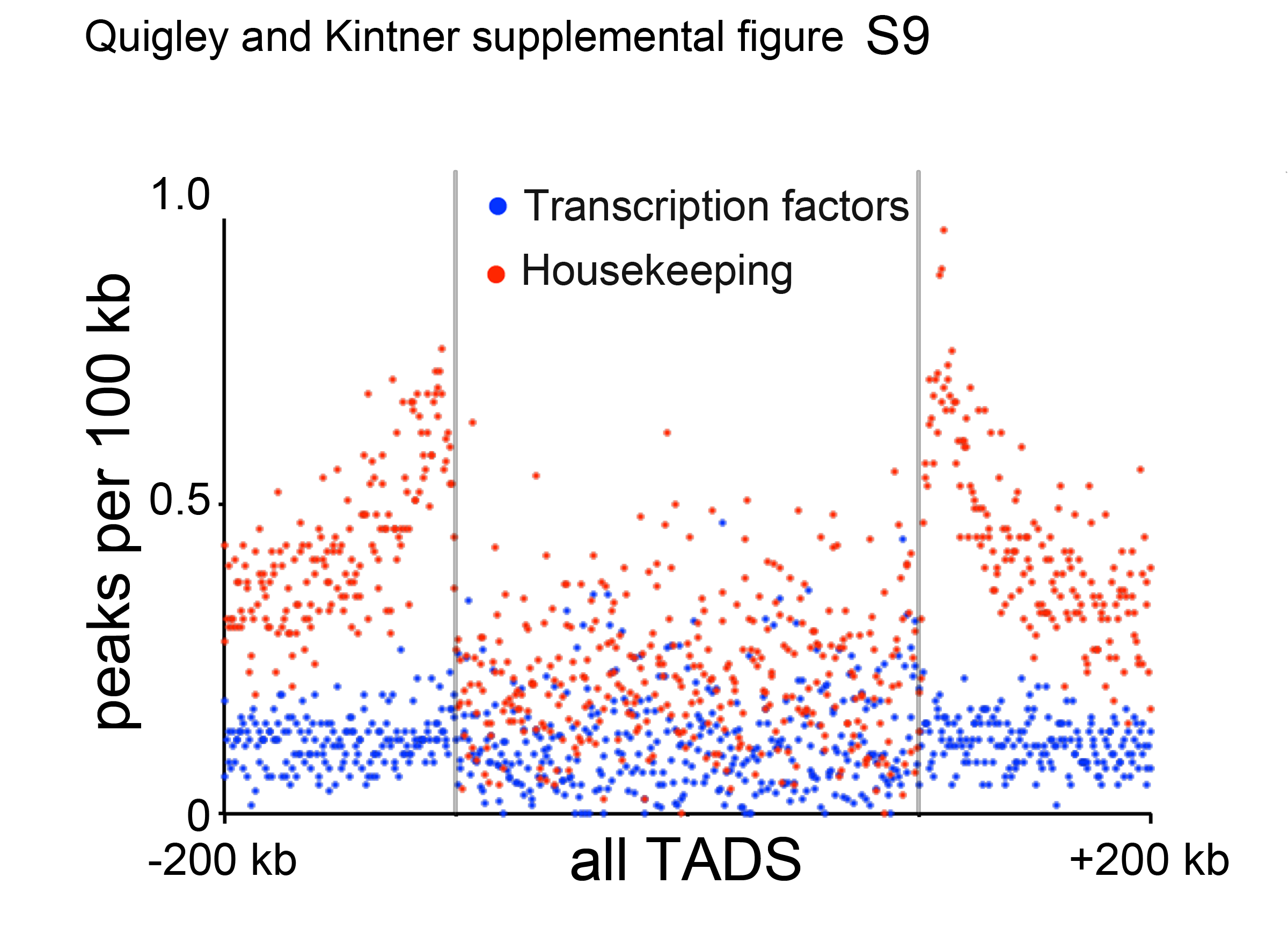

Supplement: S9 Fig — Metagene plot showing the distribution of different gene classes relative to all TADs. Domain region is in the center with boundaries marked, and all domains are normalized to the same size, whereas flanking areas are 200 kb upstream and downstream of those domain boundaries. Each quartile is broken into 175 bins, and each dot denotes one bin. (TIF) [file pgen.1006538.s009.tif]

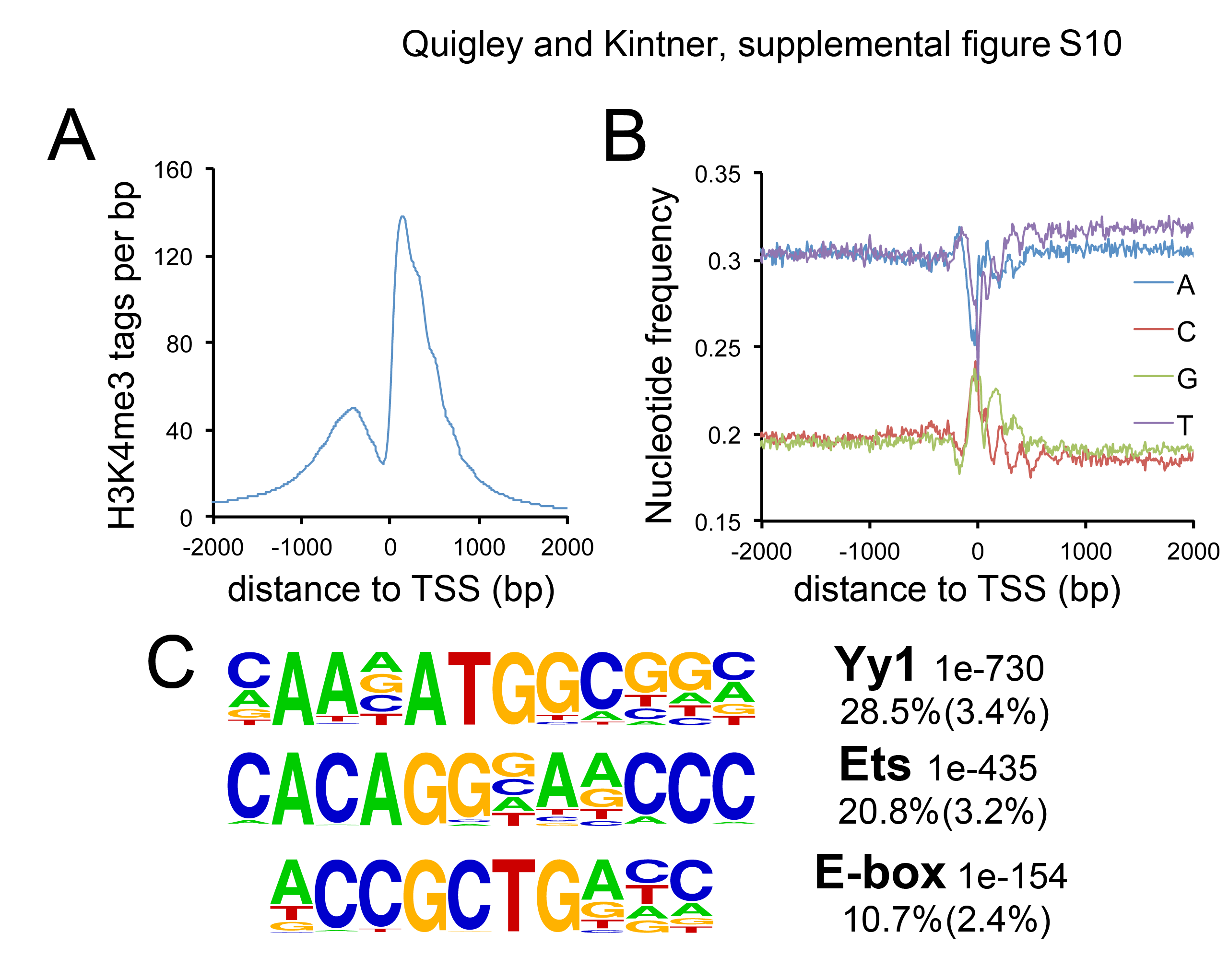

Supplement: S10 Fig — N. vectensis 5’ ends were identified by BLATing [76] all 5’ expressed sequence tags to Nve genome build v1.0 [75]. To confirm these promoters, we then overlapped them with published N. vectensis H3K4me3 ChIPseq data [77]. (A) Tag counts around peak centers of annotated Nve TSS’s. Note nucleosome-free region at center of peak. (B) Nucleotide frequencies around promoter peak centers. Note increase in GC-bias around the TSS and nucleosomal periodicity just following. (C) Top de novo motifs from H3K4me3-positive N. vectensis promoters. Note differences in core promoter motif preference relative to X. laevis, fly, and human (S6 Fig, (Louie et al., 2003; Ohler et al, 2002; van Heeringen et al., 2011). Top line of label is transcription factor family binding the motif and p-value; second line of label is frequency of motif in peaks versus background frequency (background frequency is in parentheses). (TIF) [file pgen.1006538.s010.tif]

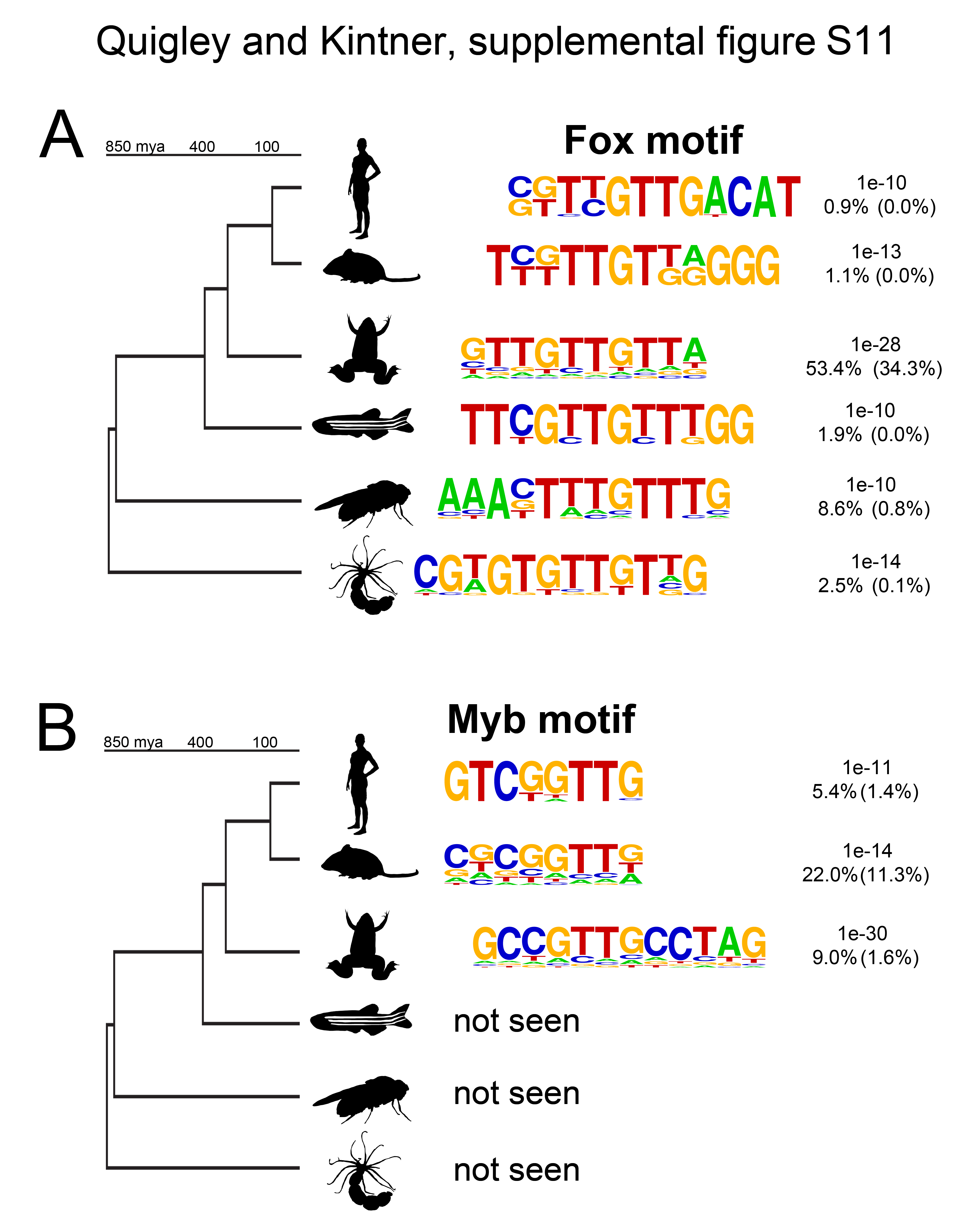

Supplement: S11 Fig — (A) Shown are the Forkhead motifs identified de novo in the promoters of MCC gene orthologs. Top line of label is p-value; second line of label is frequency of motif in peaks versus background frequency (background frequency is in parentheses). (B) Myb motifs in promoters of MCC gene orthologs. Same label convention as in (A). (TIF) [file pgen.1006538.s011.tif]

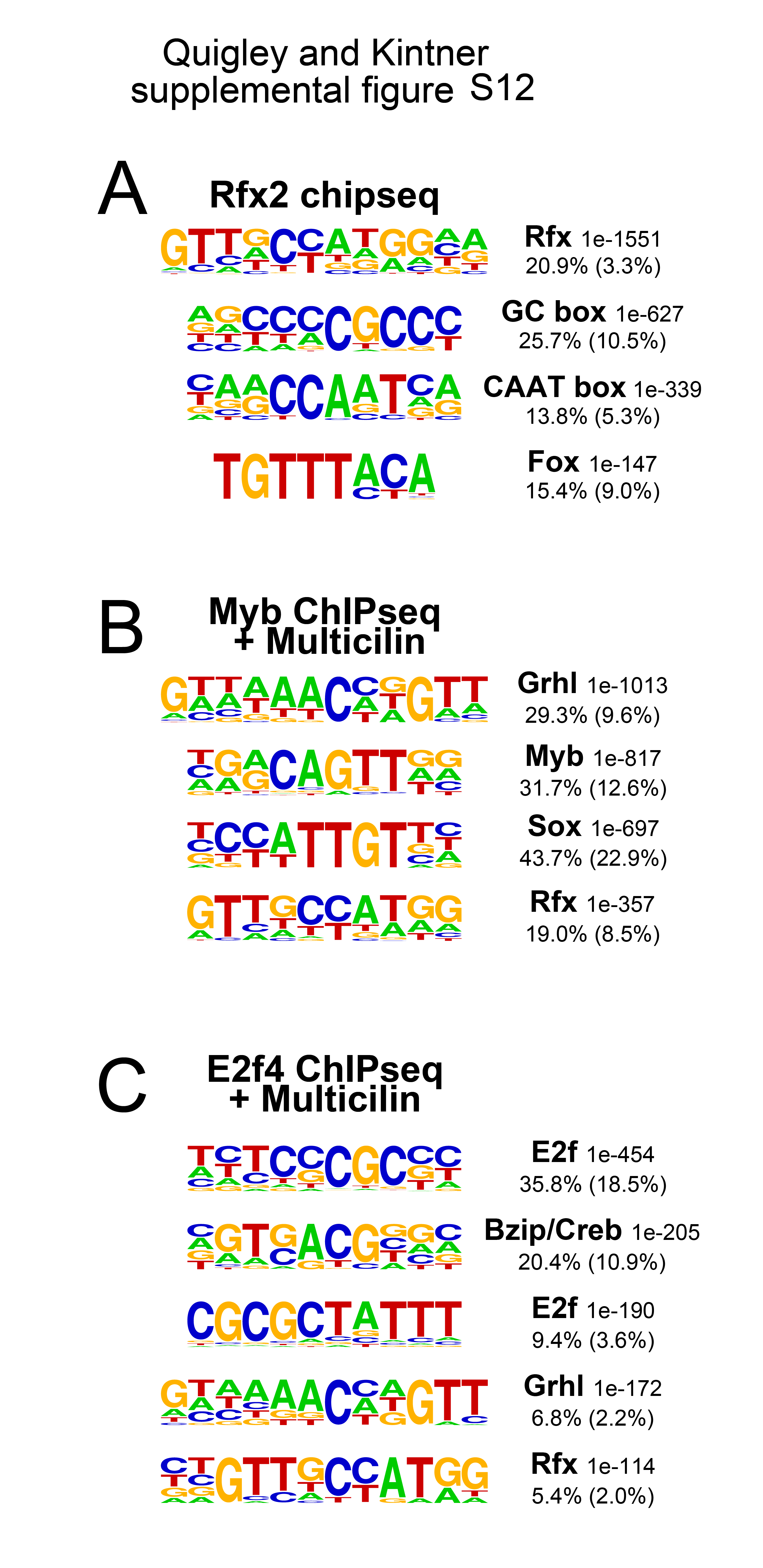

Supplement: S12 Fig — (A-C) Shown are the top de novo motifs found enriched in ChIPseq peaks of Rfx2 (A), Myb in the presence of Multicilin (B) or E2f4 in the presence of Multicilin (C). Top line of label is transcription factor family binding the motif and p-value; second line of label is frequency of motif in peaks versus background frequency (background frequency is in parentheses). Note overlap of motifs matching factors in these sets (e.g., Rfx motifs in Myb ChIPseq peaks) but reduced representation of these motifs in all promoters (S6 Fig) or all enhancers (S7 Fig). (TIF) [file pgen.1006538.s012.tif]

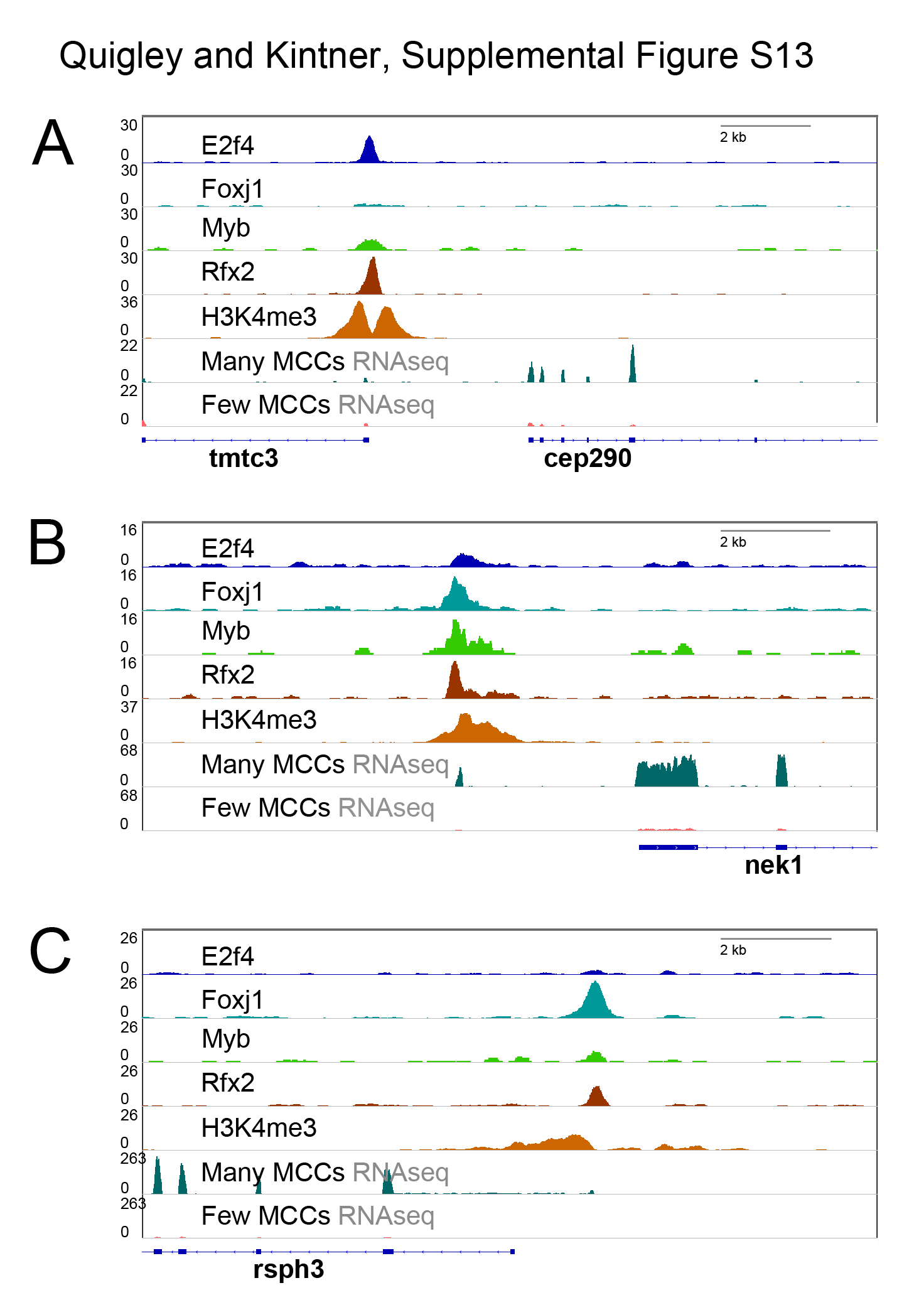

Supplement: S13 Fig — (A-C) Shown are genome browser screenshots of examples of genes with likely promoters outside a 2 kb window centered around transcriptional start sites (TSS’s from the 5’ end of the first exon of the Mayball gene models). Note both enrichment of H3K4me3 and binding of various MCC transcription factors at positions more than 1 kb upstream of described TSS. (A) A probable bidirectional promoter; note increased expression of Cep290 in MCCs but no other H3K4me3 peaks nearby. (B, C) Note upstream H3K4me3 and transcription factor peaks upstream of 5’-most annotated exons. Also note RNAseq of possible additional upstream exons. (TIF) [file pgen.1006538.s013.tif]

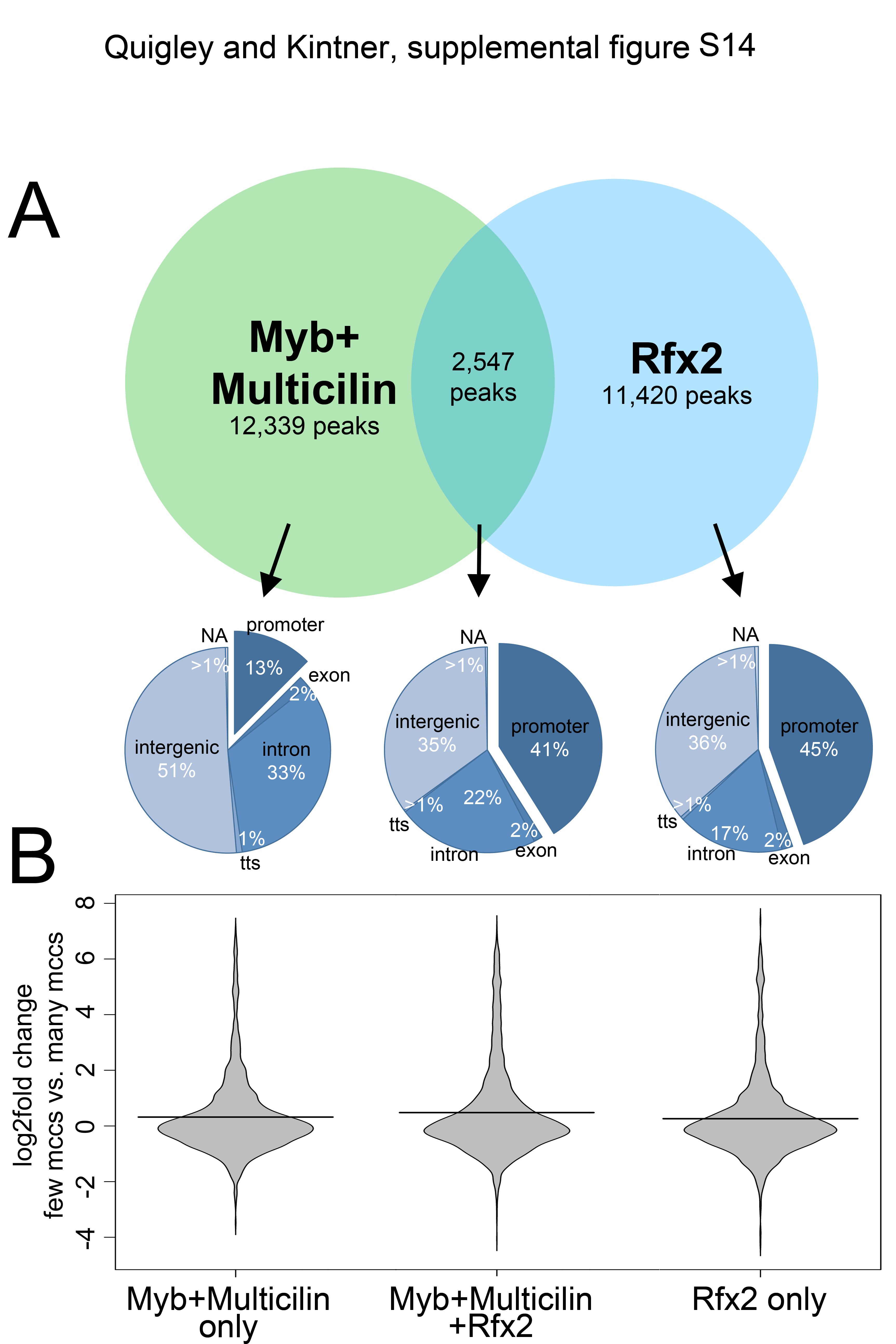

Supplement: S14 Fig — (A) Sequences were called as peaks using HOMER based on ChIPseq analysis of Rfx2 [22] or Myb in the presence of Multicilin. The top Venn diagram represents the overlap of Myb and rRx2 peak sequences, and the piecharts below show the genomic annotations of peaks bound by Myb alone, Rfx2 alone, or both Myb and Rfx2. Promoters are defined as +/- 1kb around the TSS, transcriptional termination sites (TSS) are defined as -100 bp/+1kb around the end of the transcript, and “NA” refers to genomic scaffolds containing no mapped exons. (B) Shown is a beanplot summarizing the fold change (log2) in expression at all promoters bound as indicated, based on an RNAseq analysis of progenitor manipulated to repress or promote MCC differentiation (injected with Notch-icd versus Notch-icd and Multicilin). (TIF) [file pgen.1006538.s014.tif]

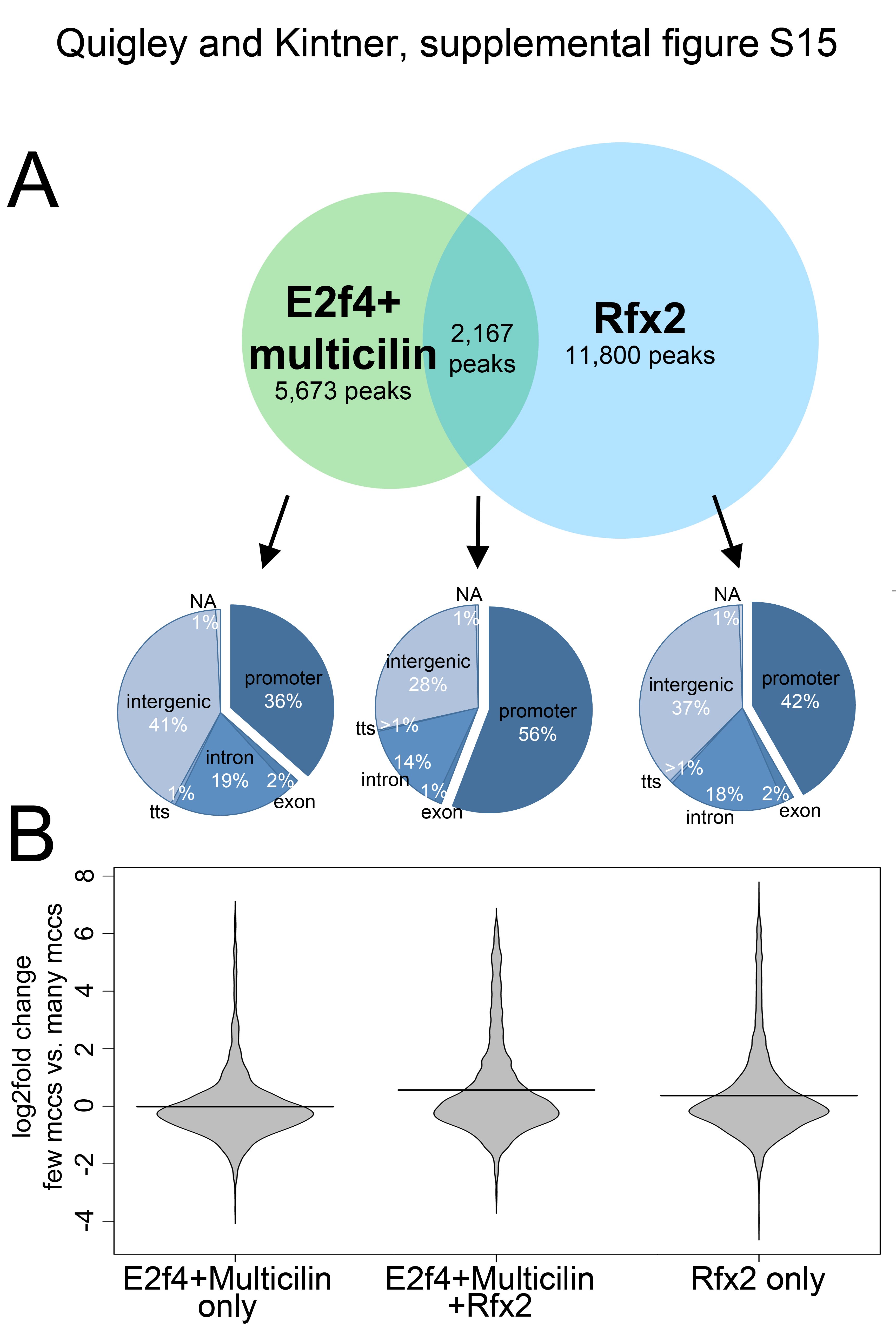

Supplement: S15 Fig — (A) Sequences were called as peaks using HOMER based on ChIPseq analysis of Rfx2 [22] or E2f4 in the presence of Multicilin [40]. The top Venn diagram represents the overlap of E2f4 and Rfx2 peak sequences, and the piecharts below show the genomic annotations of peaks bound by E2f4 alone, Rfx2 alone, or both E2f4 and Rfx2. Promoters are defined as +/- 1kb around the TSS, transcriptional termination sites (TSS) are defined as -100 bp/+1kb around the end of the transcript, and “NA” refers to genomic scaffolds containing no mapped exons. (B) Shown is a beanplot summarizing the fold change (log2) in expression at all promoters bound as indicated, based on an RNAseq analysis of progenitors manipulated to repress or promote MCC differentiation (injected with Notch-icd versus Notch-icd and Multicilin). (TIF) [file pgen.1006538.s015.tif]

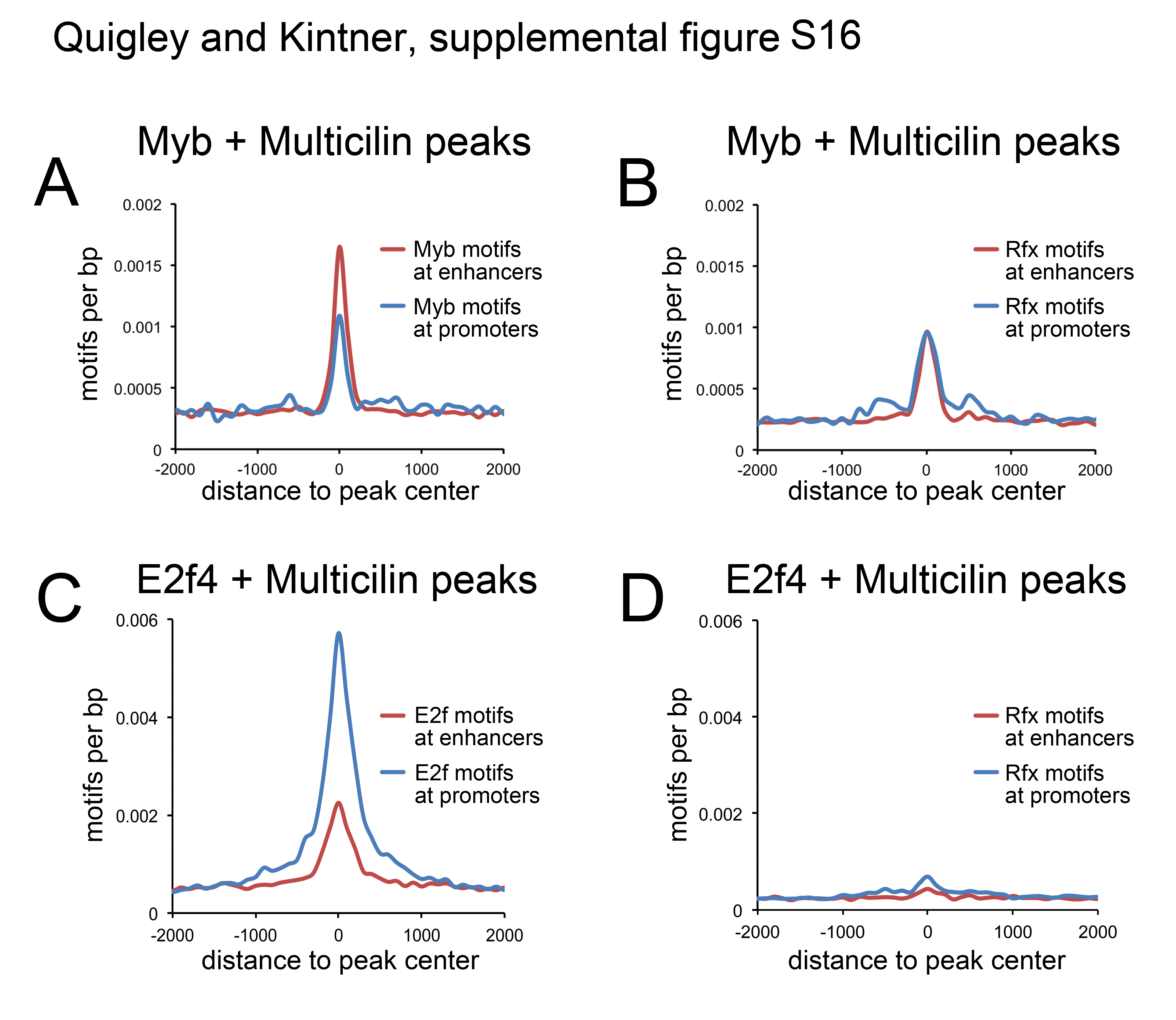

Supplement: S16 Fig — (A) Myb motifs in Myb peaks located at promoters and distal sites. (B) Rfx motifs in Myb peaks located at promoters and distal sites. (C) E2f motifs in E2f4 peaks located at promoters and distal sites. (D) Rfx motifs in E2f4 peaks located at promoters and distal sites. Note equal enrichment for Rfx motifs in Myb promoter and distal peaks, similar to Rfx motifs in Foxj1 peaks (Fig 4B). (TIF) [file pgen.1006538.s016.tif]

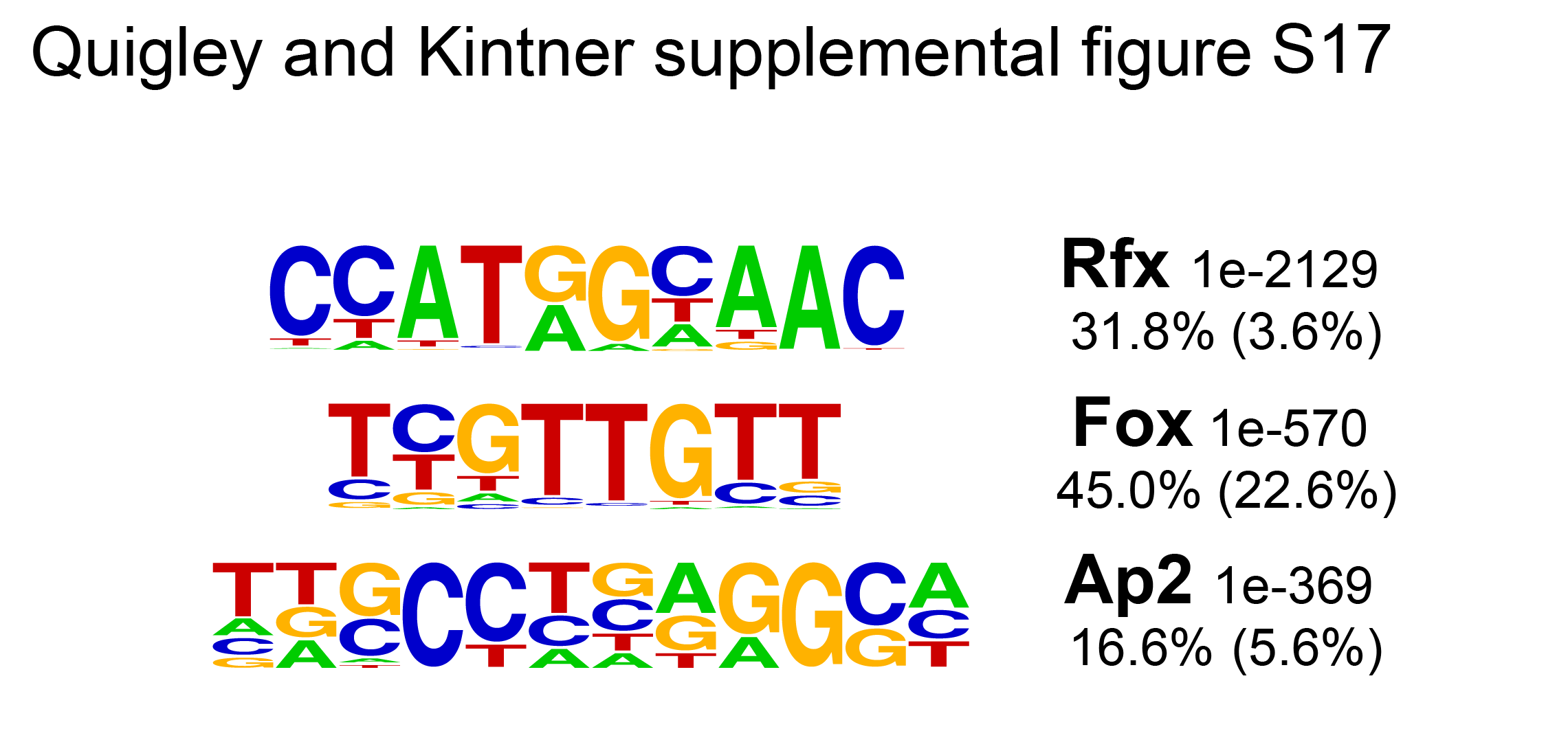

Supplement: S17 Fig — Shown are the top de novo motifs found enriched in ChIPseq peaks of Foxj1 peaks not overlapping with Rfx2 peaks. Top line of label is transcription factor family binding the motif and p-value; second line of label is frequency of motif in peaks versus background frequency (background frequency is in parentheses). (TIF) [file pgen.1006538.s017.tif]

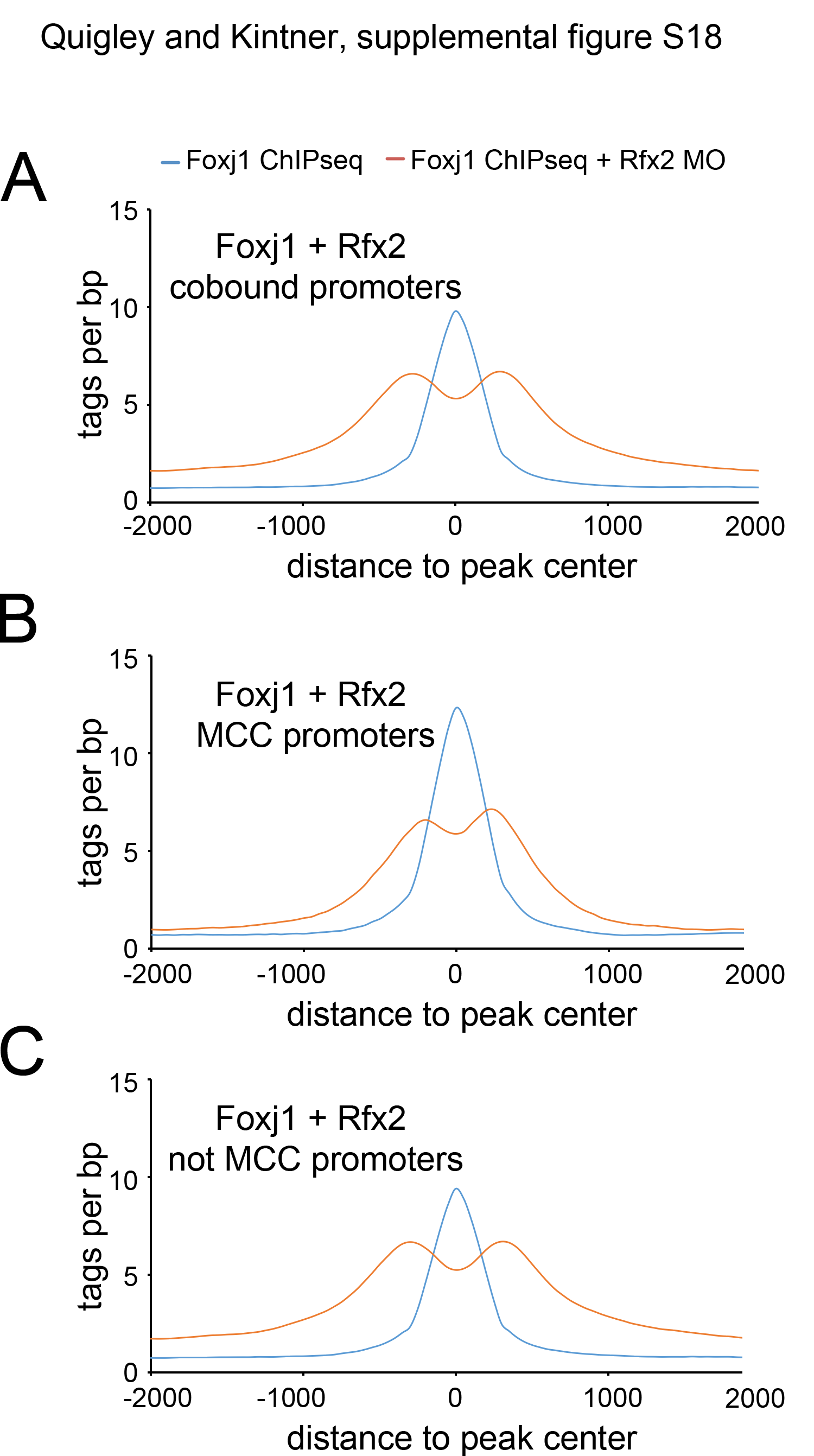

Supplement: S18 Fig — Shown are tag counts from Foxj1 ChIPseq and Foxj1 ChIPseq with Rfx2 mopholino at (A) all promoters cobound by Foxj1 and Rfx2; (B) core MCC promoters cobound by Foxj1 and Rfx2, and (C) non-MCC promoters cobound by Foxj1 and Rfx2. (TIF) [file pgen.1006538.s018.tif]

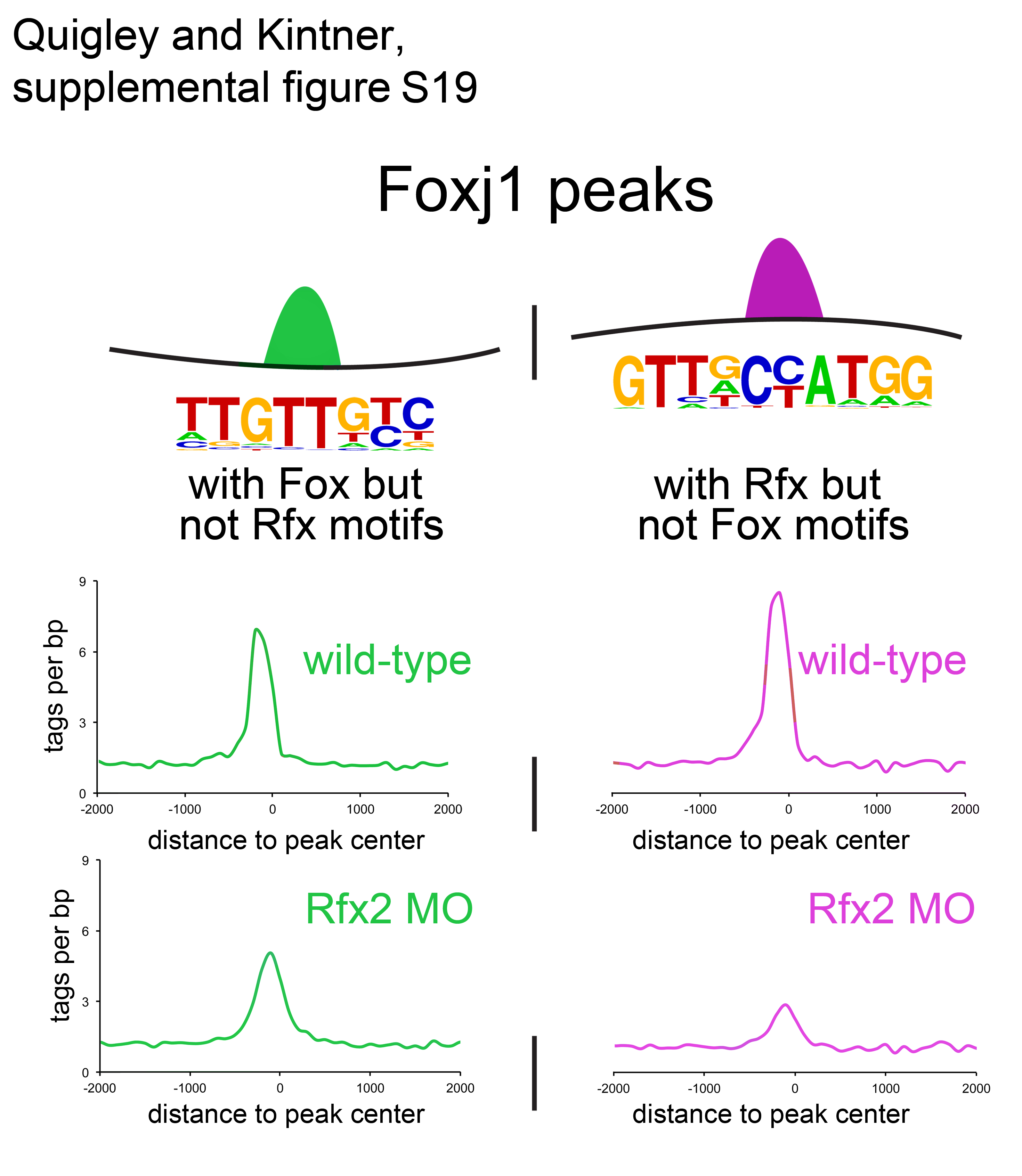

Supplement: S19 Fig — Shown are tag counts from Foxj1 ChIPseq and Foxj1 ChIPseq with Rfx2 mopholino at genomic positions with a strong Fox mot but no Rfx motif, or a strong Rfx motif and no Fox motif. (TIF) [file pgen.1006538.s019.tif]
